# Treatment of cattle with ivermectin and its effect on dung degradation and larval abundance in a tropical savanna setting

**DOI:** 10.1016/j.onehlt.2024.100950

**Published:** 2024-12-12

**Authors:** Miriam Ruhinda, Kang Xia, Cassidy Rist, Gerald Shija, Issa N. Lyimo, Felician Meza, Carlyle Brewster, Carlos Chaccour, N. Regina Rabinovich, Roger Schürch

**Affiliations:** a Department of Entomology, Virgina Polytechnic Institute & State University, Blacksburg, VA 24061, USA; b School of Plant and Environmental Sciences, Virgina Polytechnic Institute & State University, Blacksburg, VA 24061, USA; c Department of Population Health Sciences, Virgina Polytechnic Institute & State University, Blacksburg, VA 24061, USA; d Environmental Health and Ecological Sciences Department, Ifakara Health Institute, Tanzania; e Plant and Environmental Sciences Department, Clemson University, SC 29634, USA; f ISGlobal, Barcelona Institute for Global Health, Barcelona, Spain; g Universidda de Navarra, Pamplona, Spain; h Centro de Investigación Biomédica en Red de Enfermedades Infecciosas, Madrid, Spain; i TH Chan Harvard School of Public Health, Boston, USA; j Chemistry Department, College of Natural and Mathematical Sciences, The University of Dodoma, Dodoma, Tanzania; k School of Life Sciences and Bioengineering, Nelson Mandela African Institution of Science and Technology, Tengeru, Arusha, Tanzania

**Keywords:** Ivermectin, Mass drug administration, Malaria, Dung degradation, Larval abundance, Tanzania

## Abstract

When ingested as part of a blood meal, the antiparasitic drug ivermectin kills mosquitoes, making it a candidate for mass drug administration (MDA) in humans and livestock to reduce malaria transmission. When administered to livestock, most ivermectin is excreted unmetabolized in the dung within 5 days post administration. Presence of ivermectin, has been shown to adversely affect dung colonizers and dung degradation in temperate settings; however, those findings may not apply to, tropical environment, where ivermectin MDA against malaria would occur. Here we report results of a randomized field experiment conducted with dung from ivermectin-treated and control cattle to determine the effect of ivermectin on dung degradation in tropical Tanzania. For intact pats, we measured termite colonization, larval numbers and pat wet and dry weights. Pat organic matter was interpolated from a subsample of the pat (10 g wet weight). Additionally, we counted larvae growing in the treated and untreated pats in a semi-field setting. We found that termites colonized ivermectin pats more readily than controls. Despite this, wet weight decreased significantly slower in the ivermectin-treated pats in the first two weeks. As water was lost, sub-sample dry weight increased, and organic matter decreased similarly over time for the treatment and control. Interpolated for whole pats, total organic matter was higher, and larval counts were lower in the ivermectin-treated pats after the first month. Our results demonstrate an effect of ivermectin and its metabolites on dung degradation and fauna in a tropical savanna setting. Because slow dung degradation and low insect abundance negatively impact pastureland, these non-target, environmental effects must be further investigated within the context of real-world implementation of ivermectin MDA in cattle and weighed against the potential benefits for malaria control.

## 1 Introduction

Malaria is a parasitic disease transmitted by *Anopheles* mosquitoes. In 2022, malaria caused 249 million cases and 608,000 deaths, with a high burden in Africa particularly for children under 5 years and pregnant women [1]. The World Health Organization has put in place a global technical strategy targeting the reduction of malaria incidences and mortality rates by 90 % by 2030 [2] with vector control as a cornerstone approach to reaching said target [3]. Common malaria vector control strategies include the use of long-lasting insecticidal nets (LLINs) and indoor residual spraying (IRS) [4]. However, malaria-transmitting mosquitoes, have developed resistance to public health insecticides [5], and selective pressure has shifted their biting behaviors to spaces, times, and alternative blood sources not protected by LLINs or IRS including feeding outdoors, early in the evening, and upon peri-domestic cattle [6,7].

Ivermectin is an antiparasitic drug used in livestock and humans that works by blocking signal transmission in nervous systems, opening chloride channels causing hyperpolarization that results in flaccid paralysis and death in insects [8]. In mammals, the drug has an excellent safety profile [9]. Since ivermectin is effective against mosquitoes (when ingested via blood meal), but safe for mammals, it is under evaluation for mass drug administration (MDA) in humans and livestock as a potential complementary strategy to reduce malaria transmission [[10], [11], [12], [13]].

About 80 to 90 % of administered ivermectin and its metabolites in cattle are excreted in feces [14]. Dung fauna such as flies, dung beetles, termites, and others feed on excrement and help decompose pats from livestock and return nutrients to soils [15,16]. Previous studies in temperate settings have demonstrated that ivermectin and its metabolites affect dung fauna, causing delayed dung degradation [17], which could cause decreased pasture productivity and economic losses [18,19].

In contrast to temperate settings, the environmental effects of ivermectin and its metabolites in tropical settings are understudied [21,22]. It is unclear if differences in conditions such as higher sun light exposure and different climate pattern at lower latitudes [23] could mitigate the negative impact of ivermectin on dung fauna. If ivermectin MDA in cattle is determined to be effective for malaria control, it will be mostly applied in the tropical countries, making it important to understand how ivermectin treatments in cattle will affect dung fauna and degradation in tropical settings where malaria is prevalent.

Here we studied the impact of ivermectin on dung fauna and degradation in a tropical dry savanna setting in southeastern Tanzania. Specifically, we conducted qualitative observations of dung fauna colonization, and quantified termite colonization, wet weight, water content, dry weight, organic matter, and larval abundance in dung. These data will support the assessment of the environmental impacts of the drug when used for MDA in cattle for malaria vector control.

## 2 Materials & methods

We conducted the study in Sagamaganga village (8°3′50.352″ S, 36°47′46.254″ E), located in Kilombero District within Kilombero Valley, Morogoro Region, southeastern Tanzania. This valley has a wet season from February to June and a dry season from July to January with average annual temperatures between 20 °C and 32 °C [25,26,31]. The inhabitants in this valley are involved in human land use activities including livestock husbandry (e.g. cattle). However, the fieldwork was conducted during the dry season from July 2022 to October 2022 but monitored until March 2023. We selected 22 Tanzanian short horn zebu cattle from a herd that fed and grazed in the same area. These were allocated 1:1 to the control and ivermectin treatment groups using a random computer-generated sequence in R. Cattle ranged in age from 1 to 4 years, weighed between 90 and 351 kg, and had not been exposed to antiparasitic drugs in the preceding three months. A veterinarian checked the cattle before the study, qualifying them as physiologically mature. Similar studies have used cattle of different age ranges [17,19,20]. We had 11 cattle (8 males and 3 females) in the control group and 11 ivermectin treated cattle (7 males and 4 females). The control group had weight of (186 ± 93.5) kg and age (2.0 ± 1.1) years while the ivermectin group weighed (148 ± 45.7) kg and aged (1.5 ± 0.5) years. We employed a randomized controlled experimental design to evaluate ivermectin's effect on dung degradation and fauna.

We used IVOMEC® which is 1 % ivermectin injectable formulation (1 ml = 10 mg of active ingredient) commonly used in most rural villages for routine deworming of livestock including cattle. We administered ivermectin (200 μg/kg body weight) subcutaneously to 11 cattle on 8th July 2022. The cattle owners provided consent for the treatment and agreed to respect withdrawal periods for milking and/or slaughter. All procedures were conducted according to the local veterinary practices and approved by the Ifakara Health Institute Institutional Review Board (IHI/IRB/No:30–2022), and the National Institute for Medical Research in Tanzania (NIMR/HQ/R.8a/Vol.IX/4159). The National Institute for Medical Research in Tanzania also granted permission to publish the research findings (Ref.No.BD.242/437/01B/61).

### 2.1 Data collection

#### 2.1.1 Dung degradation study

For the dung degradation study, on each day, 22 replicate pats were prepared for each treatment group, making a total of 44 pats per day (22 control and 22 treated) and 220 pats in total for 5 collection days. This was achieved by collecting two fresh dung pats daily per treatment from each of the 22 cattle, mixing the two pats from each individual in the grazing field, and then homogenizing the mixture to create 1 kg samples. These homogenized pats were then randomly distributed in a designated field separate from the grazing area. We placed pats 2 m apart on a plastic net (8–10 mm mesh) to facilitate retrieval and covered them with a chicken wire mesh cage to exclude birds but not insects.

On days 15, 30 and 45 in the field, we first measured wet weight of each entire pat, and then randomly scooped 10 g from its surface for further analysis of organic matter content. These 10 g sub-samples were sent to the Ifakara Health Institute laboratory, where they were refrigerated for 5 to 7 days at 4 °C until further analysis. 110 treatment and 110 control samples were collected on those specific days. Termite infestation observations were also done on day 15 and 30 for each pat.

For processing, 10 g sub-samples were homogenized by grinding them with a mortar and pestle. We put the 10 g sub-samples in a crucible which we previously weighed to the nearest 0.01 g. We then dried the sub-samples in the oven for 6 h at 110 °C to remove all moisture content [28]. After drying, the sub-samples in the crucibles were cooled in a desiccator for 30 min before weighing to prevent moisture absorption. We calculated the percentage dry matter as the remaining weight of a sub-sample after drying [28].

We determined organic matter in the dung pat using the loss on ignition method also known as ashing which helps separate mineral content (ash) from organic constituents that are susceptible to decomposition [29] by: heating the oven dried samples from the previous step (sub samples heated at 110 °C that were cooled and weighed) in a pre-heated furnace at 500 °C overnight (12h) and then cooled them for 30 min before weighing. We then calculated organic matter content by determining percentages from the differences in weight of subsamples at 500 °C and at 110 °C [18,29,30].

Percent LOI (ploi) was calculated as:

$$\text{ploi}=100\times \frac{\left(\mathrm{dwc}110-\mathrm{dwc}500\right)}{\mathrm{dw}110}$$

where *dwc*110is the crucible and sample weight at 110 °C, *dwc*500 is the crucible and sample.

Weight at 500 °C, and *dwc*110is the original dry weight of the sample as obtained from the previous step.

From the sub-sample organic matter that we obtained above, we extrapolated to the full pat to obtain the total organic matter in the individual pats by multiplying wet weight of the sub-samples and the lost organic matter.

Total organic matter=wetweight×(dwc110−dwc500)/10.

where *dwc*500 is the crucible and sample weight at 500 °C and *dwc*500 is the crucible and.

sample weight at 110 °C.

#### 2.1.2 Qualitative insect observations in the field

We used standard methods to assess dung insect larval abundance [[33], [42]]. In this case fresh dung for this experiment was collected on the same dates from the control and treated animals and counts performed on larvae. For the larval study, on day 3, 10 and 29 after ivermectin administration, we haphazardly collected dung from ivermectin and untreated cattle as they were deposited in the field. We then mixed the individual pats thoroughly. From the collected pats, we made 1 kg standardized treated and control composite pats (*N* = 10 each) and placed them on a wire mesh in the field to aid recovery. Following a 1 day field exposure period, we collected pats and placed them in an emergence cage to recover insects [34]. We recorded visitations of arthropods to the dung and documented beetle larval counts for the larval study and termite presence in the two treatments for the degradation study. Termites were only observed and recorded in the pats that were used in the dung degradation study.

##### 2.1.2.1 Insect abundance under semi-field dung conditions

Due to the hot temperature in the field, dung pats dried out quickly and we could not record adult insect emergence. To try and obtain insect data we modified the experiment to a semi-field system. Therefore, we enriched the samples with a second set of 10 (5 treated and 5 control) random pats on day 1, 2, 3 and 5 after administration and left the pats out to be colonized by insects for one day on a mesh and then transferred them to containers housed in a semi-field system that had a screen house enclosure. We counted and recorded the number of larvae found in dung pats on a weekly basis and observed their development until the pats dried up in the semi-field system.

### 2.2 Statistical analysis

We used R version 4.2.1 (R Core Team and others 2020) for all statistical analyses. To account for the longitudinal nature of our data with repeated measures of individual pats, we used mixed models to study the effect of ivermectin treatment on wet weight, water content, dry weight, organic matter, termite infestation and larval abundance. In these models with the three degradation outcomes as response variables, we added random intercepts for individual dung pats, and we added treatment × time interactions as fixed effects [35]. Similarly, we used Poisson mixed models to study effects the ivermectin treatment on larval abundance. Here, we used larval counts as our response variable and accounted for the source of the data (sets 1 and 2, see above) with random intercepts and treatment, scaled days and scaled days squared as fixed effects. The latter was done to account for the initial increase and then decrease of new larvae emerging. All mixed models were implemented using the lme4 package [36]. We used the emmeans package to extract estimated marginal means and compute standard errors and confidence intervals [37].

For the dung degradation study, all 220 pats (samples) were measured. Pat loss occurred on day 15 for 2 samples (one control and one ivermectin treated pat) due to termite infestation and on day 30 for 3 ivermectin samples because of oven malfunctioning. Because these failures were in a small part of the sample (i.e., < 5 %), and because they were also largely balanced between treatments, these pats were treated as missing in the data set.

To determine whether termite infestation affected the degradation results or not, we conducted a sensitivity analysis excluding any termite infested pats. The results were comparable to the full analysis set, and we therefore only show the results from the full analysis set.

## 3 Results

### 3.1 Qualitative observations of timing and presence of fauna

In the first few hours after dung was deposited in the field, we observed Diptera, mainly Calliphoridae and Muscidae. While dung pats formed a dried crust on the upper side, they remained moist on the underside where they were in contact with soil. Colonization was followed by Coleoptera mainly Scarabaeidae and Staphylinidae. These were present until pats dried 2 to 7 days after they were deposited in the field, and then they were followed by Isoptera. Hymenoptera (family of Formicidae, mostly safari ants *Dorylus* sp.), Collembola and Acari were also observed on the dry pats.

As the pats dried, they became less attractive to most arthropods. Qualitatively, ivermectin treated pats also lost moisture more slowly as compared to control pats. Overall, we observed fewer Coleoptera larvae in ivermectin pats.

### 3.2 Ivermectin treated pats were more likely to be infested with termites

**Fig. 1:**
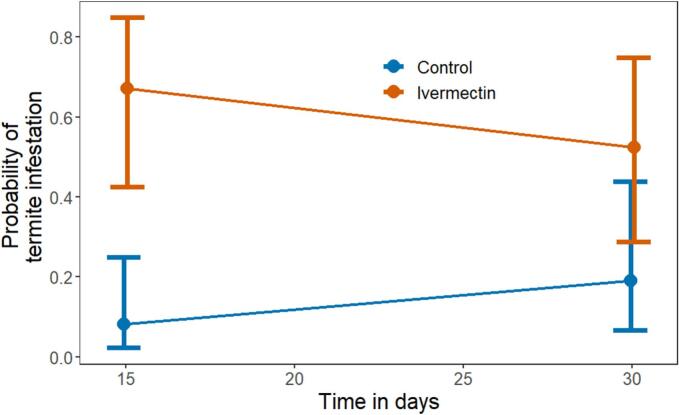
Probability of termite infestation and 95 % confidence intervals after 15 and 30 days. Termites significantly preferred ivermectin treated pats compared to control pats on day 15, but not on day 30. (*n* = 220).

Ivermectin treated pats were significantly more likely to be infested by termites on day 15 (odds ratio: 23.2 (3.9 to 136.2); df = ∞; *z* = −3.48; *p* < 0.001), but not on day 30 (odds ratio: 4.7 (0.9 to 23.3); df = ∞; *z* = −1.89; *p* = 0.059; see Fig. 1). The point estimate for the probability of termite infestation in ivermectin pats decreased from 15 to 30 days (*p* = 0.484), while the reverse occurred in control pats, though neither was significant (*p* = 0.142).

### 3.3 Wet weight decreased more slowly in ivermectin treated pats, irrespective of termite presence

**Fig. 2:**
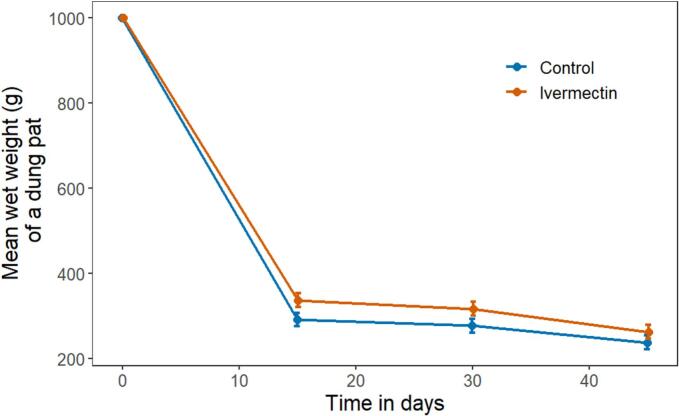
Mean wet weight and 95 % confidence intervals of whole pats over time. Overall, ivermectin treated pats displayed significantly slower wet weight decrease in comparison to control, with the largest effect occurring from day 0 to 15. (*n* = 220).

Both treatment and control wet weights decreased from Day 0 to Day 30. By Day 15, there was a significant effect of treatment, as control pats were 13.4 % lighter than treatment pats (control pats = 291.9 g (275.7 to 308.1) versus treatment pats = 337.2 g (321.0 to 353.3), mean difference: −45.3 g (68.2 to −22.4); df = 435.1; *t* = −3.89; *p* < 0.001, Fig. 2). The difference was 12.4 % by Day 30 (control pats = 277.8 g (261.6 to 294.1) versus treatment pats = 317.0 g (300.7 to 333.2), mean difference: −39.2 g (−62.1 to −16.2); df = 437.8; *t* = −3.35; p < 0.001). The difference was also maintained to a smaller, non-statistically significant, extent (9.3 %) on Day 45 (control pats = 238.5 g (222.2 to 254.7) versus treatment pats = 262.9 g (246.7 to 279.2), mean difference: −24.5 g (−47.4 to −1.5); df = 437.8; *t* = −2.09; *p* = 0.037); see Fig. 2).

We tested whether termites affected the wet weight decrease, but we did not find a relationship between termite presence and wet weight decrease on Day 15 (controls: 11.1 g (−7.8 to 30.0) ivermectin: 20.3 g (2.8 to 37.9); mean difference: −9.2 g (−35.0 to 16.6); df = 214; *t* = −0.7; *p* = 0.482).

### 3.4 Water content decreased similarly in treatment and control pats

**Fig. 3:**
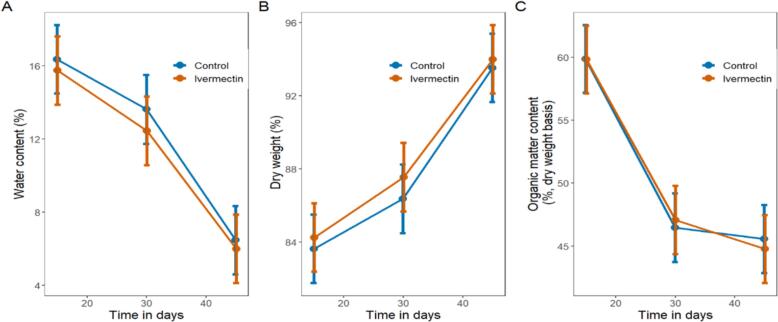
Change of percent water, dry weight and organic matter content of subsamples. **A** Water content (%) and 95 % confidence interval over time. Ivermectin did not affect water content on day 15, 30 and 45 and the ivermectin and control pats lose water content on similar trajectories.(*n* = 220) **B** Dry matter content (%) and 95 % confidence intervals over time. Ivermectin did not affect dry weight content on day 15, 30 and 45. These results show that ivermectin and control pats dry weight content increase similarly.(n = 220) **C** Organic matter content and 95 % confidence intervals over time. Ivermectin did not affect organic matter content, on day 15, 30 and 45. These results show that organic matter in sub-samples decreased similarly in ivermectin and control pats.(n = 220).

Water content decreased over the three time points for both treatment and control dung pats. The treatments did not differ with respect to water content on day 15 (control pats = 16.4 % (14.5 to 18.2) versus treatment pats = 15.7 % (13.9 to 17.6), mean difference: 0.6 % (−2.0 to 3.3); df = 643.9; *t* = 0.47; *p* = 0.639, day 30 (control pats = 13.6 % (11.7 to 15.5) versus treatment pats = 12.4 % (10.6 to 14.3), mean difference: 1.2 % (−1.5 to 3.8); df = 644.1; *t* = 0.88; *p* = 0.381) nor day 45 (control pats = 6.5 % (4.6 to 8.3) versus treatment pats = 6.0 % (4.1 to 7.9), mean difference: 0.5 % (−2.2 to 3.1); df = 643.9; *t* = 0.35; *p* = 0.724); see Fig. 3A).

### 3.5 Dry weight content changes were similar for treatment and control pats

Dry weight content of sub-samples increased over the three time points for both treatment and control dung pats. The control and ivermectin treated pats did not differ on day 15 control 83.6 % (81.8 to 85.5); ivermectin: 84.3 % (82.4 to 86.1); mean difference: −0.6 % (−3.3 to 2.0); df = 643.9; *t* = −0.47; *p* = 0.639), day 30 control: 86.4 % (84.5 to 88.3); ivermectin: 87.6 % (85.7 to 89.4); mean difference: −1.2 % (−3.8 to 1.5); df = 644.1; *t* = −0.88; *p* = 0.381), or day 45 control: 93.5 % (91.7 to 95.4); ivermectin: 94.0 % (92.1 to 95.9); mean difference: −0.5 % (−3.1 to 2.2); df = 643.9; *t* = −0.35; *p* = 0.724; see Fig. 3B).

### 3.6 Organic matter content decreased similarly between ivermectin-treated and control pats

There was no effect of treatment on organic matter content. Ivermectin treated pats and control pats did not differ at day 15 (control: 59.9 % (57.2 to 62.6); ivermectin: 59.8 % (57.1 to 62.5); mean difference: 0.0 % (−3.8 to 3.8); df = 631.8; *t* = 0.02; *p* = 0.983), day 30 (control: 46.5 % (43.7 to 49.2); ivermectin: 47.1 % (44.4 to 49.8); mean difference: −0.6 (−4.5 to 3.2); df = 632.8; *t* = −0.31;*p* = 0.758), or day 45 (control: 45.6 % (42.9 to 48.3); ivermectin: 44.8 % (42.1 to 47.5); mean difference: 0.8 % (−3.0 to 4.6); df = 632.1; *t* = 0.41; *p* = 0.682; see Fig. 3C).

### 3.7 Total organic matter/pat decreased slower in ivermectin pats

**Fig. 4:**
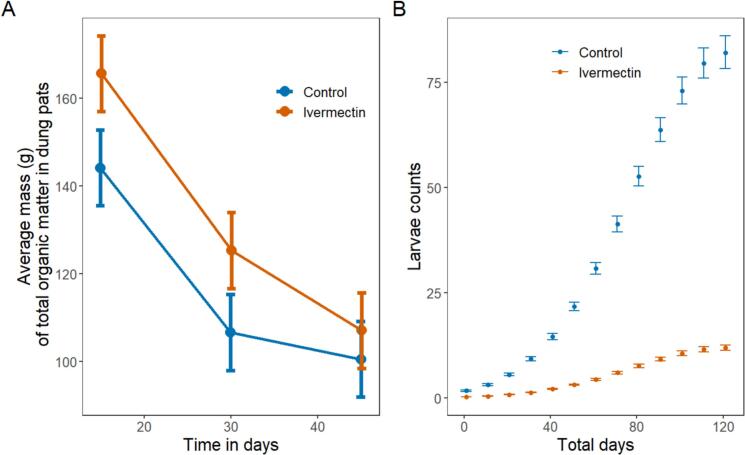
**A** Mean mass of total organic matter/pat and 95 % confidence interval over time. Ivermectin affects the average mass of total organic matter on day 15, 30 and 45 in the dung pats.(*n* = 220) **B** Mean larvae count and 95 % confidence interval over time. There were fewer larvae in ivermectin treated pats in comparison to control. These results are significant and show that ivermectin negatively impacts larvae abundance. (*n* = 40).

Both treatment and control total organic matter/pat, as interpolated from subsamples to whole pats, decreased from Day 0 to Day 30. There was a significant effect of treatment on total organic matter remaining in the pats, with control pats having approx. 13.2 % lower mass, or an estimated mass at 144.1 g (135.5 to 152.7) versus treatment pats at 166.1 g (157.5 to 174.7). This is a mean absolute difference of −22.0 g (−34.1 to −9.8; df = 563.5; *t* = −3.55; *p* < 0.001). By day 30 control pats had approx. 14.7 % less mass, or 106.8 g (98.1 to 115.5) remaining of controls, compared to 125.2 g (116.5 to 133.8) of ivermectin treated pats for a mean absolute difference of −18.4 g (−30.6 to −6.1; df = 568.1; *t* = −2.93; *p* = 0.003). On day 45, this was no longer significant (control pats approx. 6.2 % lower mass; control pats: 100.5 g (91.9 to 109.2); ivermectin pats: 107.1 g (98.5 to 115.7); mean difference: −6.6 g (−18.8 to 5.6); df = 564.9; *t* = −1.06; *p* = 0.291, Fig. 4A).

### 3.8 Ivermectin treated pats had fewer larvae compared to control pats

We found a significant effect of ivermectin treatment on larvae counts, with the difference between treatment and control increasing over time (Fig. 4B). There was a significant effect of treatment on larvae population from day 1 to day 124, (mean counts on day 124 (95 %): control pats = 82.1 (78.1 to 86.2), treatment pats = 11.9 (11.3 to12.6), corresponding to an increase by 689 % (666 to 712); df = ∞; z = 112.92; *p* < 0.001).

## 4 Discussion and conclusion

We determined the impact of ivermectin MDA in cattle on dung degradation and dung fauna in a tropical dry savanna setting in Tanzania, focusing on dung degradation in terms of wet weight, dry weight, loss of organic matter and larval abundance. In our study, ivermectin treated pats degraded more slowly in terms of wet weight (Fig. 2) and the total organic matter/pat (Fig. 4A). Additionally, we found that more beetle larvae lived in control than ivermectin treated pats. Originally, we aimed to count adults that would have emerged from dung in the two groups. Intense hot weather in the field led to a modification of the study to a semi-field setting for further observation. During the single day in the field, adult insects visited the pats. By the end of the day, the pats had started drying out, particularly on the surface, which had become crusty. Once moved to the semi-field setting, we periodically counted larvae in the pats. Interestingly, we observed only beetle larvae and no fly larvae. The intense weather conditions and the drying dung could likely be unsuitable for fly larvae survival. We continued counting larvae until the pats completely dried out. However, no adult insects emerged during the experiment.

Together, our results suggest that ivermectin hinders colonization of dung by some arthropods mainly Coleoptera, affecting their abundance, and ultimately slowing dung degradation in tropical savanna settings.

Wet weight of whole pats decreased in the treatments by a difference of 14 % in 15 days, 12 % by day 30 and 9 % by day 45. Dry weight of sub-samples increased (Fig. 3B) and water content decreased, presumably because of moisture loss associated with dry weather for our study area [39]. There was no statistically significant treatment difference in the organic matter content among the sub-samples. However, extrapolating the organic matter to whole pats, we found significant differences in the first month of degradation, but not the period after that. This extrapolation has uncertainties as insects moved the pats and some left hollow spaces in the dung pats. In these cases, less dung or soil remained in the samples. Still, a sensitivity analysis restricted to pats without termite infestation upheld the main result. In summary, we find these data to be compelling evidence that ivermectin given to cattle decreases dung degradation in the field of a tropical environment [23,40]. A number of studies have found similar results in the temperate settings [17,21,27,33,34]. The similarity is somewhat surprising, as ivermectin degrades quickly in sunlight [14] and metabolites like 3′′-*O*-demethylivermectin (3DI) go below the threshold for detection on the upper dung layer in a week's time post dung placement [24]. Despite the higher solar irradiation at the lower latitudes, ivermectin and its metabolites must have had an effect on dung before their degradation in our study.

We observed termites throughout the dry season, corroborating previous observations that point to their ability to tolerate harsh dry conditions [43,44]. Termites specifically add soil contents in dung during mound making [45] facilitating microbial activity. Termites also help organic matter break into nutrients taken up by plants [46]. Despite this important role of termites in dung degradation, there was no association between termite infestation and dung degradation, measured in terms of wet weight, in this study. Maybe such an effect would become evident after the 45 days that we allotted to our experiment. Surprisingly, termites preferentially associated with ivermectin-treated pats. This was a novel observation that echoes a similar report on dung beetles [[47], [48], [49]]. Wardhough and Manon suggested that some volatile metabolites of ivermectin in the dung as well as changes in the gut flora of cattle caused by ivermectin may be involved in the bias to ivermectin-treated dung [50]. However, a recent study found no evidence that dung undergoes direct changes in the volatile compounds releases or if there is a change in gut microbiota in cattle [51]. In our study, given the overall lower rates of colonization by insects, perhaps the termites face less competition in the ivermectin- treated pats. Further research on the phenomenon is needed. We did not observe adult helminths in any dung pats.

While less obvious in our study, colonization of pats with other dung organisms may also be crucial. For example, beetles remove dung [8], and adult beetles consume the liquid portion of dung, but their survival seems to be unaffected by ivermectin with further evidence of past exposures not impacting the future ecosystem at the area of exposure [52]. Although as dung organisms make their movements, they facilitate drying [48,53] and decomposition [54]. Less larval activity in ivermectin pats is problematic since larvae contribute to dung degradation by direct decomposition of organic matter, mixing, aerating, and breaking dung into smaller particles enabling microbial decomposition [41,55]. The number of larvae in dung increases with time; however, in the field, their numbers quickly decreased as dung lost its moisture in the tropical dry season tropical setting [56], explaining their inability to survive [57]. In our study, adult Diptera were only observed on the surface of dung pats for one day, when they were still moist on the top layer. Afterwards, conditions for Diptera development were not favorable [58]. Adult beetles, on the other hand, seemed less affected, but the lower larvae counts in ivermectin treated pats in the semi-field experiment points to a reduction in the ability to produce viable offspring under these conditions [[32], [38], [59]].

We conducted our study during the dry season and loss of dung diversity due to dry conditions may exacerbate the effects of ivermectin during that time [60]. On the other hand, increased ivermectin photodegradation may have lessened the impact of ivermectin treatments on dung degradation [24]. There is a critical need to conduct similar studies during wet season in the tropics to rule out the dry condition effect on microbial and insect activity and investigate further the ivermectin effect during this period crucial to malaria spread. Furthermore, it is unknown what level of difference in dung degradation rate is environmentally relevant and may lead to downstream effects like pasture fouling due to prolonged presence of dung pats, particularly in the extensive livestock management systems found in tropical climates. Despite these limitations, our study represents an important first step if we want to evaluate the environmental impact of mass drug administration in cattle to curb the spread of malaria in humans.

In summary, results from tropical Tanzania add to the previous findings from the temperate region studies which demonstrate that ivermectin negatively affects dung degradation and larval development. Therefore, if ivermectin MDA in cattle proves to be an effective method for malaria control, its non-target environmental consequences should be further quantified and evaluated using a One Health approach to better account for the full costs and benefits of such a control strategy.

## Author contribution

*N. Regina* Rabinovich, Carlos Chaccour, Kang Xia, Cassidy Rist, Roger Schürch, Carlyle Brewster, Issa N. Lyimo: Conceptualisation, Funding acquisition. Kang Xia, Cassidy Rist, Issa N. Lyimo, Roger Schürch: Supervision and oversight. Miriam Ruhinda, Felician Meza and Gerald Shija: Data collection. Miriam Ruhinda, Roger Schürch: Methodology, Data curation, Data analysis, Writing of original draft. All authors: Review & editing, approval of the final version.

## CRediT authorship contribution statement

**Miriam Ruhinda:** Writing – review & editing, Writing – original draft, Visualization, Validation, Project administration, Methodology, Investigation, Formal analysis, Data curation. **Kang Xia:** Writing – review & editing, Supervision, Project administration, Methodology, Funding acquisition, Conceptualization. **Cassidy Rist:** Writing – review & editing, Supervision, Project administration, Methodology, Funding acquisition, Conceptualization. **Gerald Shija:** Writing – review & editing, Methodology, Investigation. **Issa N. Lyimo:** Writing – review & editing, Supervision, Project administration, Investigation, Funding acquisition, Conceptualization. **Felician Meza:** Writing – review & editing, Investigation. **Carlyle Brewster:** Writing – review & editing, Methodology, Funding acquisition, Conceptualization. **Carlos Chaccour:** Writing – review & editing, Project administration, Funding acquisition, Conceptualization. **N. Regina Rabinovich:** Writing – review & editing, Project administration, Funding acquisition, Conceptualization. **Roger Schürch:** Writing – review & editing, Visualization, Validation, Supervision, Resources, Project administration, Methodology, Investigation, Funding acquisition, Formal analysis, Data curation, Conceptualization.

## Declaration of competing interest

The authors declare no conflict of interest.

## Data Availability

This work was funded by Unitaid through the BOHEMIA project (https://bohemiaconsortium.org/). The funding source had no role in study design, data collection and analysis, decision to publish, or preparation of the manuscript. The BOHEMIA consortium has agreed to make the data underlying each manuscript openly available upon publication. The data supporting this paper can be found in the Virginia Tech research repository.

## Appendix A Supplementary data

Supplementary information for Field-Based Studies on Insect and Dung Interaction Image 1
