# Supplementary material for: Treatment of cattle with ivermectin and its effect on dung degradation and larval abundance in a tropical savanna setting
Source: One Health. 2024 Dec 12;20:100950. doi: 10.1016/j.onehlt.2024.100950 (PMC11699432; doi:10.1016/j.onehlt.2024.100950)
Supplement: Supplementary file 1 — Supplementary information for Field-Based Studies on Insect and Dung Interaction [file mmc1.docx]

**Supplementary information**


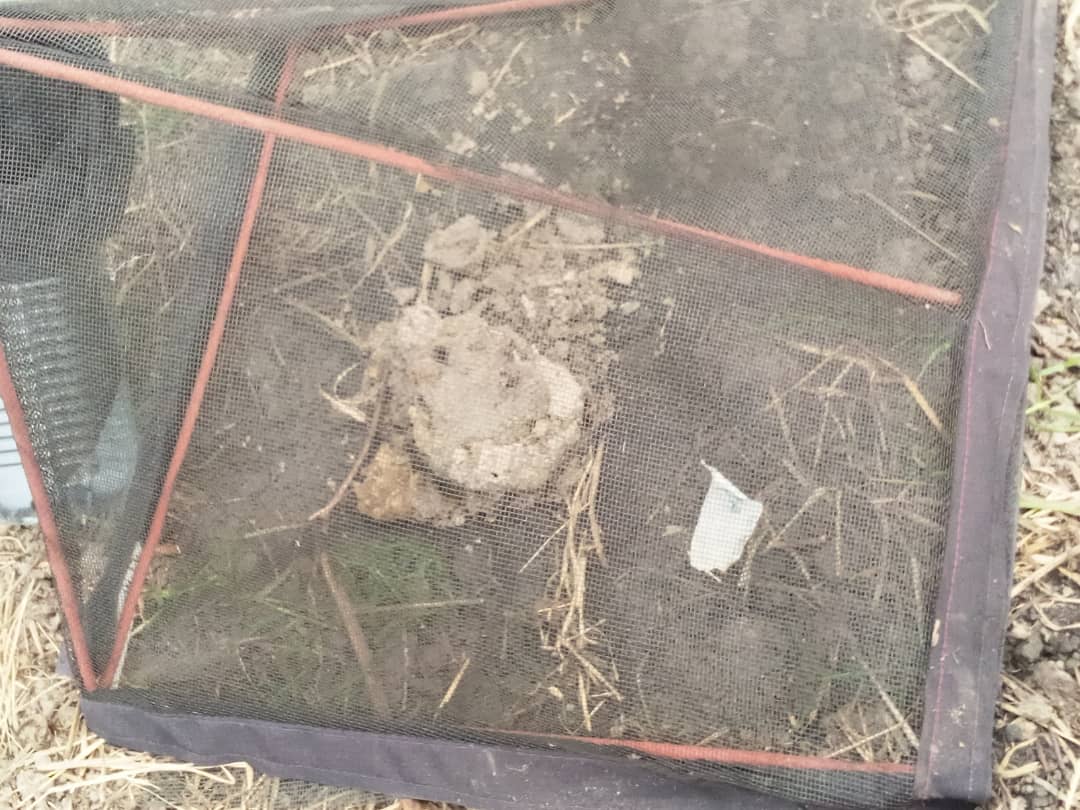


Supplementary figure 1: Emergence cage in the field


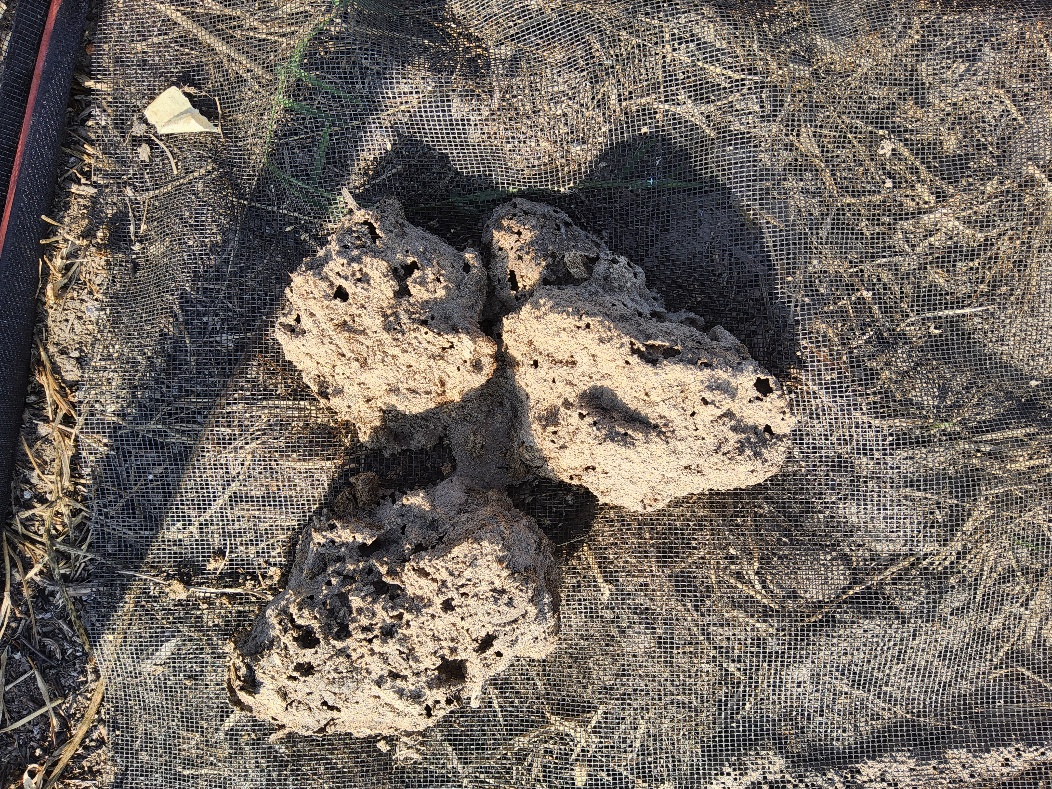


Supplementary figure 2: Termite infestation on dung
